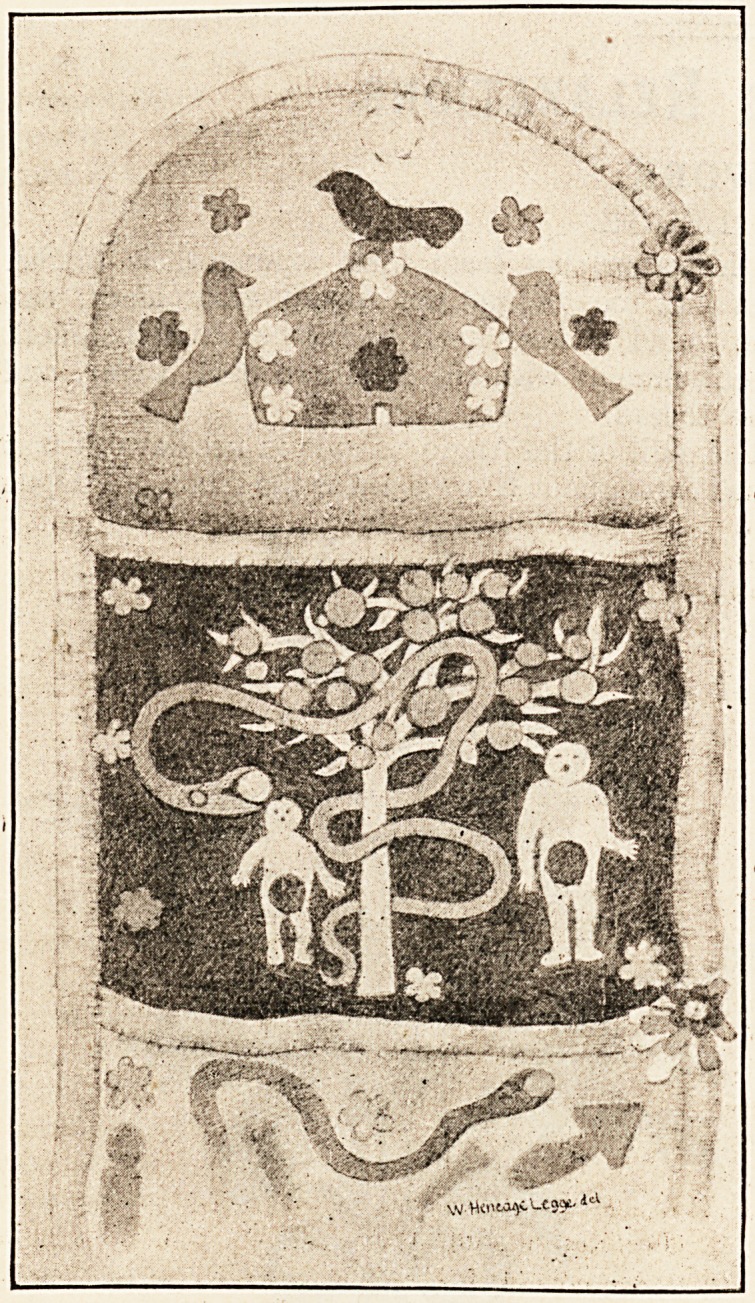# Old Needlework

**Published:** 1910-04-09

**Authors:** W. Heneage Legge


### The Practitioner's Relaxations.

### OLD NEEDLEWORK.

By W. HENEAGE LEGGE.

Among the varied subjects that may attract the attention and affection of the collector, old needlework and embroidery is by no means the least interesting. The antiquity of the art is possibly as prehistoric as pottery, that most ancient of the handicrafts of man, and which, though entirely utilitarian in origin and intent, did not despise decoration, nay, continually attempted it. It is indeed worthy of notice that some of the oldest and most primitive pottery patterns—if forms so simple deserve the designation—took the shape of stitches in regular arrangement in rows or bands.

The man—perhaps, rather, the woman—of the early Stone Age who first adopted skins, more as a protection against weather and climate than as a cover of nakedness, doubtless used a whole hide, skewered around the body with pins of thorn or bone. But even so primitive a being must soon have seen the possibility of cutting and shaping the hide and joining edge to edge with stitches of some tough twisted fibre or thin thong carried through the material by a needle of bone or ossified tendon. With such coarse methods stitches would be only too apparent to the eye, and any regularly-repeating form or pattern they assumed would soon suggest ornamental use, even to an eye untutored in art or not consciously concerned with beauty. Hence we find the form of "herring-bone" stitch, so much used to strengthen and disguise a join or seam, reproduced on earthenware pots and pans of the most ancient times, and doubtless transferred by impression of the fabric on the still-soft clay.

At the earliest historic period embroidery had attained a position among the handicrafts of man. Homer makes frequent allusions to the art, of which doubtless he had seen much in Asia Minor, where there flourished from an ancient age an embroidery enriched with gold thread and precious stones in many an aspect of domestic life, from costly hangings of houses to the embellished harness of horses.

Among the Egyptians and other ancient folk the art was freely practised, as we know not only from the historian, but from finds among their tombs. Weaving, too, though not strictly embroidery, was soon found to lend itself to further embellishment by enrichment with the needle in gold, silver, and brass thread.

The skill of the Anglo-Saxons in needlework was famed throughout Europe under the name of "Opus

Anglicanum." Some have conjectured that the celebrated Bayeux tapestry was worked by women of the conquered race. However this may be, it is very remarkable that so momentous an episode of history should find its most enduring contemporary record not in inscriptions on stone or metal, or in tough parchments, but in so delicate and perishing an art as needlework. All through the Middle Ages, the Renaissance, and even the unrestful times of "the Great Rebellion," needlework formed the chief employment of the women of those days; and much beautiful work was produced in wall-hangings, bed-furniture, and bookbindings. Ancient wills constantly contain bequests such as "curtains of green linen broidered with silver swans," or "hangings of blue cloth sown with golden lilies."

With the "advance of civilisation" the invention of much machinery, the introduction of wall paper, and the general supervention of utilitarian ideas, this art fell into some disuetude. Nevertheless, it was not dead, and there were not wanting those who strove to awaken and encourage it. Thus we come across in not a few eighteenth-century wills charitable bequests of money for the teaching and advancement of children in "the use of the needle."

Such examples of early needlework as survive are not common, and they bring high prices when they come into the market. Specimens of later date may be met with in various domestic articles, as fire-screens, "cosies," and "tidies." Of the latter the one illustrated here is noteworthy as the work of a soldier about the time of the Waterloo campaign. It consists of several pockets one above another, the whole 24 inches long and $4\frac{3}{4}$ inches broad. The devices are many and curious, and are carried out in appliqué work in flannel, red and white designs on a blue ground, white and blue on a red, blue and red on a white. But the workmanship is very unorthodox, the devices being affixed more by tacking than by the proper appliqué stitch. The top compartment shows Noah's Ark in red, with the raven on top in blue, flanked by the two doves in red. The first pocket is more elaborate, and shows on a blue ground the Tree of Knowledge, with the Serpent intertwined in red, Adam and Eve standing on each side, "charged" with blue fig-leaves. Other compartments contain the Ladder of Life, the Cross-keys, Skull and Cross-bones, etc. It is difficult to see what object the little piece of needlework shown in the other illustration could have been designed to serve, other than to depict a beautiful group of flowers with the needle, for it is worked most delicately with silk on a piece of thin paper. On the back is written "Marguerita Perugini fecit."

But the particular product of the "use of the needle" which the collector of moderate means is most likely to meet with is some specimen of the once common "sampler." There is a peculiar charm in these quaint productions, part of which doubtless is due to the archaic appearance which their straight lines and abundant angles give them, part to their colouring, and part, possibly, to the associated ideas which they raise in the mind, mental pictures of some rural "dame's school" wherein little hands, with busy fingers, patiently worked out their alphabet, perchance with fear and trembling, wondering meanwhile as to the inner meaning of the somewhat metaphysical mottoes which occupied some important position in these simple but pleasing works of art.

## Figures and Tables

**Figure f1:**
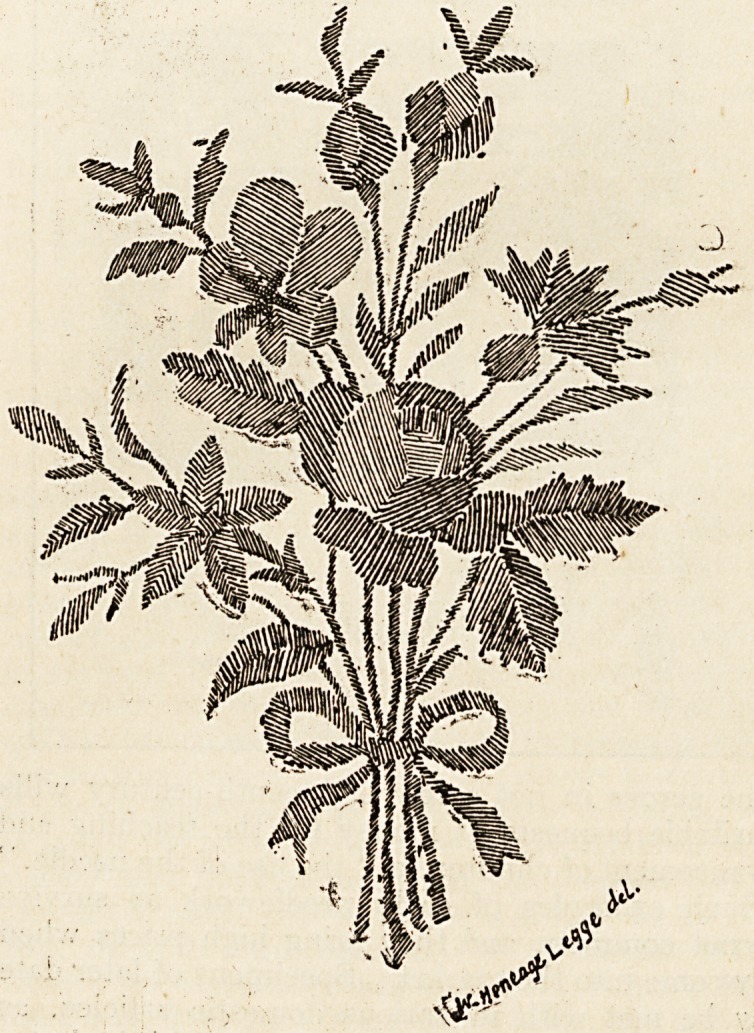


**Figure f2:**